# The Investigation of Adoption of Voice-User Interface (VUI) in Smart Home Systems among Chinese Older Adults

**DOI:** 10.3390/s22041614

**Published:** 2022-02-18

**Authors:** Yao Song, Yanpu Yang, Peiyao Cheng

**Affiliations:** 1 College of Literature and Journalism, Sichuan University, Chengdu 610064, China; yao.song@scu.edu.cn; 2 Digital Convergence Laboratory of Chinese Cultural Inheritance and Global Communication, Sichuan University, Chengdu 610207, China; 3 School of Construction Machinery, Chang’an University, Xi’an 716604, China; yangyanpu@chd.edu.cn; 4 Design Department, School of Social Science and Humanity, Harbin Institute of Technology (Shenzhen), Shenzhen 518055, China

**Keywords:** older adults, senior technology adoption model (STAM), technology adoption model (TAM), trust voice-user interface (VUI)

## Abstract

Driven by advanced voice interaction technology, the voice-user interface (VUI) has gained popularity in recent years. VUI has been integrated into various devices in the context of the smart home system. In comparison with traditional interaction methods, VUI provides multiple benefits. VUI allows for hands-free and eyes-free interaction. It also enables users to perform multiple tasks while interacting. Moreover, as VUI is highly similar to a natural conversation in daily lives, it is intuitive to learn. The advantages provided by VUI are particularly beneficial to older adults, who suffer from decreases in physical and cognitive abilities, which hinder their interaction with electronic devices through traditional methods. However, the factors that influence older adults’ adoption of VUI remain unknown. This study addresses this research gap by proposing a conceptual model. On the basis of the technology adoption model (TAM) and the senior technology adoption model (STAM), this study considers the characteristic of VUI and the characteristic of older adults through incorporating the construct of trust and aging-related characteristics (i.e., perceived physical conditions, mobile self-efficacy, technology anxiety, self-actualization). A survey was designed and conducted. A total of 420 Chinese older adults participated in this survey, and they were current or potential users of VUI. Through structural equation modeling, data were analyzed. Results showed a good fit with the proposed conceptual model. Path analysis revealed that three factors determine Chinese older adults’ adoption of VUI: perceived usefulness, perceived ease of use, and trust. Aging-related characteristics also influence older adults’ adoption of VUI, but they are mediated by perceived usefulness, perceived ease of use, and trust. Specifically, mobile self-efficacy is demonstrated to positively influence trust and perceived ease of use but negatively influence perceived usefulness. Self-actualization exhibits positive influences on perceived usefulness and perceived ease of use. Technology anxiety only exerts influence on perceived ease of use in a marginal way. No significant influences of perceived physical conditions were found. This study extends the TAM and STAM by incorporating additional variables to explain Chinese older adults’ adoption of VUI. These results also provide valuable implications for developing suitable VUI for older adults as well as planning actionable communication strategies for promoting VUI among Chinese older adults.

## 1. Introduction

Supported by advanced voice interaction technology, interacting with products through speech is no longer a scenario in fiction. It now happens in our daily lives. When planning for the next day, we can simply ask a smart speaker what the weather will be. It will search for weather information automatically and respond to us by speech with the temperature and probability of rain tomorrow. It can even provide recommendations for bringing an umbrella or wearing a coat. VUI has gained its popularity in this decade along with the dramatic improvements of relevant technologies. With the improvements of speech recognition technology, VUI-embedded systems can recognize voice commands accurately. For example, Google has announced that their speech recognition accuracy rate has reached 95% [1]. Natural language processing (NLP) has also been largely improved and it enables VUI-integrated systems to be capable of interpreting the intended meanings of users. As a result, an increasing number of electronic devices integrate voice-user interfaces (VUI), such as virtual assistant Siri developed by Apple, smart speaker Google Home, and Echo launched by Amazon.

Different from traditional interaction ways that require input and output devices [2], VUI allows users to interact with electronic devices through speech. Users can command electronic devices by talking to them, similar to a natural conversation in daily lives. Such hands-free interaction provides huge benefits. As input devices such as keyboard and mouse are no longer necessary, users can interact with devices in a shorter time. The interaction speed can be largely improved [3]. Moreover, as VUI allow users’ eyes and hands to be free while interacting, users can complete multiple tasks [4]. For instance, users can search for information through VUI while driving a car, which can improve driving safety [5]. Because of the advantages of voice interaction, VUI has been adopted at a fast rate [6]. In particular, VUI has become an important modality for smart home systems. Various smart home devices integrate VUI, such as smart speakers, cleaning robots, and smart television. The integration of VUI in smart home systems largely improves interaction efficiency. For instance, instead of using a remote controller, users can directly talk to a smart television. In comparison with using a remote controller through pressing buttons, voice commands largely reduce interaction time and improve interaction accuracy.

VUI can be particularly beneficial for older adults in smart home contexts. As older adults often suffer from the gradual loss of physical and cognitive capabilities, interacting with devices through traditional graphical user interfaces (GUI) can be difficult [7]. Older adults may have problems reading texts on the screens. They may also fail to type or click due to shaky hands. Instead, VUI can be a promising solution. As speech is a natural method of interpersonal communication, VUI can be much easier for older adults to learn and operate [8,9,10]. However, given the potential benefits brought by VUI, how older adults perceive VUI remains unclear. Fragmented evidence shows that older adults are open to VUI embedded in smart speakers [8,9,11] but they also show concerns in adopting them [9]. Therefore, it is necessary to understand what factors drive older adults’ adoption of VUI.

The size of the older population is increasing worldwide every year. This phenomenon is even more serious in China. The number of older adults, whose ages are above 60, has reached 2.6 million, occupying 18.7% of the general population in China [12]. The percentage of older adults has increased by 5.44% in comparison with 2010. These changes will make profound consequences for Chinese society. Older adults are more easily suffering from chronic disease [13]. Due to the decline of physical and cognitive abilities, older adults may encounter difficulties in daily lives and thus become less independent, which burden families and societies. The adoption of new technology becomes a promising way to improve older adults’ well-being, such as maintaining independence, improving their safety, and being active in their social networks [14,15].

However, although adopting technologies can assist older adults’ daily livers, they often show resistance to adopting new technologies in comparison with young people [16,17,18,19,20]. Such resistances can become even stronger with the increase in ages [21,22]. Additionally, there is no exception for VUI adoption. Thus, it is crucial to understand how Chinese older adults perceive VUI and what factors influence their adoption of VUI. Gaining these insights can help companies to develop or adapt the current VUI in order to fulfill the needs of the senior segment [23].

This study aims to fill in this gap. Specifically, this study investigates the factors that influence Chinese older adults’ adoption of VUI. Through literature review, this study firstly proposes a conceptual framework with eight variables that determine Chinese older adults’ adoption of VUI. Next, a survey was designed and conducted with 420 valid participants. Data analyses were conducted using structural equation modeling.

## 2. Literature Review

This research aims to investigate older adults’ adoption intention of VUI in China. To investigate users’ adoption of VUI, the theoretical models related to technology adoption are reviewed. On the basis of current technology adoption models, the characteristic of VUI is specifically considered. As VUI can threaten users’ privacy, users have to trust VUI systems in order to use them effectively. Thus, we include trust as an additional factor and review relevant theories. Furthermore, as we especially target older adults, we integrate aging-related characteristics into the framework. The literature related to aging-related characteristics is reviewed.

### 2.1. The Theoretical Models Related to Technology Adoption

To understand the driving factors of users’ adoption of technologies, several theoretical frameworks have been proposed. Roger [24] has proposed the diffusion of innovation model, which posits five factors that influence diffusion: complexity, tribality, observability, compatibility, and relative advantages. Davis [25] proposed the technology acceptance model (TAM), which suggests that users’ adoption intention of technology is mainly influenced by the perceived usefulness and perceived ease of use of the technology. The features of the technology itself largely determine users’ perceived usefulness and perceived ease of use. Some subsequent models have also been proposed by incorporating social norms (TAM2) [26] and enjoyments (TAM3) [27]. Extending the TAM, Venkatesh et al. [28] further established the unified theory of acceptance and use of technology (UTAUT), which pointed out that technology adoption is primarily influenced by effort expectancy, performance expectancy, social influence, and facilitating conditions. The diffusion of innovation model has been recommended for use in commercial contexts and for predicting organizational adoption of innovation [29]. TAM and UTAUT are considered to be more proper for explaining individuals’ adoption of technology [30,31].

Although TAM and UTAUT are robust and powerful models to predict users’ adoption of technology, the explanatory powers differ from contexts [32,33]. TAM and UTAUT are also found to carry some limitations to explain users’ adoption of new technologies [34]. In order to improve explanatory powers of explaining users’ adoption of technology in specific contexts, new constructs have been identified and included in TAM and UTAUT [35,36,37]. For instance, Wang, Tao, Yu and Qu [38] extended the UTAUT by including the additional factors of technology characteristics and task characteristics to explain Chinese users’ acceptance of healthcare wearable devices. To understand users’ adoption of digital voice assistants, Fernandes and Oliveira [39] extended the TAM by considering the influence of trust, social interactivity and social presence. Therefore, although TAM is a robust model to explain users’ adoption of technology adoption, it needs to be adjusted depending on specific contexts.

### 2.2. Trust and Technology Adoption

To understand users’ adoption of information technology-related applications, previous studies pointed out the uncertainty of the IT environment [40]. Thus, it is necessary to incorporate the construct of trust into the extended versions of TAM and UTAUT [41,42,43,44]. Trust is a multidimensional concept [45]. Mayer et al. [46] proposed the three dimensions of trusts: (1) competence, which indicates the skills and capabilities that allow a system to perform effectively; (2) benevolence, which refers to one’s willingness to believe that another party will not make use of its vulnerability; and (3) integrity, which is defined as one’s subjective evaluation of the appropriateness of another party’s behavior. When used in different contexts, the construct of trust can be interpreted in different ways. In the contexts of users’ adoption of new technology, trust mainly captures the ability dimension and it refers to individuals’ subjective evaluation of the reliability, functionality and helpfulness of the technology [47]. In e-commerce contexts, where transactions occurred, trust reflects the dimension of benevolence and integrity. Trust is defined as one’s belief that the e-commerce systems will behave responsibly [46,48].

In the context of users’ adoption of VUI, the dimension of benevolence and integrity of trust can be more prominent. Specifically, while using VUI, users have to allow systems to record and track their speech in order to improve VUI system accuracy [49]. The VUI system records the users’ voice command as well as the background sound in order to provide immediate feedback [50]. Due to this, users may feel risky or even threatened while using VUI systems to some extent. In this case, users’ trust reflects their perception of VUI systems’ willingness to behave in a socially responsible way: the VUI systems will not leak or misuse their personal information, and their personal information is protected by the VUI systems. Previous research has demonstrated the significant influence of trust on users’ adoption in various contexts, such as e-commerce [51], 5G technology [52], Internet banking [53], digital voice assistants [39] and young people’s adoption of VUI [40]. Therefore, to understand older adults’ adoption of VUI, this study includes the construct of trust.

### 2.3. Older Adults’ Technology Adoption

To explain the technology adoption of a specific user group, previous studies found that the TAM and UTAUT may be insufficient [54]. Specifically, the models used for young users can be insufficient for older adults because the two groups value different facets of technology. Older adults show resistance to adopting new technologies. Such resistances come from different sources, including physical and psychological factors. A number of studies have demonstrated that older adults can encounter more difficulties while adopting technologies due to the decline of physical capabilities, such as the gradual loss of sensorial capabilities of vision and hearing [55] and dexterity problems which cause difficulties in typing [56]. Psychological factors can also cause problems for older adults to adopt new technologies [57,58,59]. For instance, in comparison with young people, older adults are found to suffer more from anxiety when adopting technologies.

In order to gain a comprehensive understanding of older adults’ adoption of technology, the senior technology acceptance model (STAM) has been proposed [60], which highlighted the importance of aging-related characteristics. Specifically, STAM extends TAM through incorporating self-efficacy, technology anxiety and facilitating conditions. Results demonstrated the significant influences of aging-related characteristics on older adults’ adoption. Similarly, to understand the factors that influence older adults’ adoption of mobile health in China, Deng, Mo and Liu [61] also considered the aging-related characteristic, including perceived physical condition, technology anxiety, self-actualization needs and resistance to change. Therefore, it is necessary to include aging-related characteristics to better understand older adults’ adoption of VUI.

**Figure 1:**
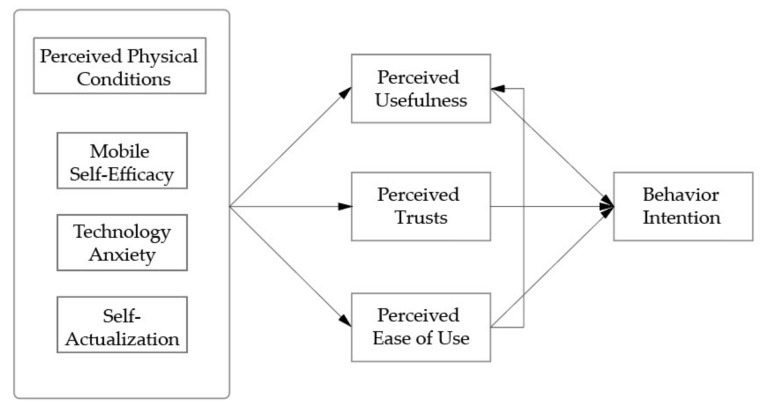
The conceptual framework of this study.

In summary, this study aims to understand the factors that influence Chinese older adults’ adoption of VUI. Although user adoption of VUI has been investigated [40], it targeted the young generation in western contexts. Limited research attention has been paid to understanding older adults’ adoption of VUI in Chinese contexts. This study aims to fill in this gap. To do so, this study starts from TAM and considers the uniqueness of VUI by including the factor of trust. Next, by referring to the STAM and other studies related to aging characteristics, this study integrates four ageing-related characteristics (i.e., mobile self-efficacy, technology anxiety, self-actualization, physical health condition). The research framework is shown in Figure 1.

## 3. Hypothesis Development

### 3.1. VUI and Technology Acceptance Model

According to the TAM [25], users’ adoption of new technology is predicted by perceived ease of use (PEOU) and perceived usefulness. When encountering a new technological application, users tend to subjectively assess the efforts required for using it (PEOU) and the benefits gained from using it. Extensive research demonstrates that users’ intention of adopting new technological applications is positively related to PU and PEOU [62,63,64,65]. PU largely results from PEOU. In other words, when users perceive a technological application as difficult to use, their perception of users can also be largely discounted. TAM considers technology characteristics as external variables that influence PU and PEOU.

In the context of users’ adoption of VUI, PU refers to the utilitarian benefits of using VUI-driven systems, whereas PEOU reflects users’ perceived difficulty of learning to use VUI. UI directly measures the extent to which users’ intention of using VUI. VUI allows users to complete various interaction tasks by voice controls rather than visual interface controls [66]. Thus, VUI provides multiple benefits, such as remote interaction and multiple task interaction. Moreover, compared with the traditional user interface, VUI enables users to interact with smart devices in an intuitive way: talking to the smart devices as if talking to a real person. Therefore, considering the benefits brought by VUI, we expect that the utilitarian benefits and convenience will positively influence users’ usage intention. In addition, as interaction with devices through VUI is highly similar to an interpersonal conversation in daily lives, it should be intuitive to learn. Effortless learning can further improve users’ perceptions of usefulness. Previous studies have demonstrated the positive relationships between PU, PEOU and UI [67] as well as the positive links between PEOU and PU [68]. The following hypotheses are given:

**Hypothesis** **H1-1:** *Perceived usefulness positively influences behavioral intention*.

**Hypothesis** **H1-2:** *Perceived ease of use positively influences behavioral intention*.

**Hypothesis** **H1-3:** *Perceived ease of use positively influences perceived usefulness*.

### 3.2. Trust and Technology Adoption

As discussed earlier, trust is an essential factor in influencing users’ adoption of technology. VUI has to record users’ voice command and their daily speech to be responsive. In other words, users have to share their speech in order to use VUI effectively and efficiently. Users may feel more risks associated with using VUI than using a traditional user interface. In this case, trust means that users believe that their personal information will be protected during the usage of VUI [40]. In users’ adoption of VUI, trust helps alleviate users’ concern that their personal information has been shared and might be misused [40]. Therefore, we expect that trust is positively related to older adults’ adoption intention of VUI.

**Hypothesis** **H2:** *Trust positively influences behavioral intention*.

### 3.3. Perceived Physical Conditions

To better understand older adults’ adoption of VUI, it is necessary to consider the changes caused by aging [60,61]. Specifically, in this study, four aspects related to aging were considered: perceived physical conditions, mobile self-efficacy, technology anxiety and self-actualization.

#### 3.3.1. Perceived Physical Conditions

Perceived physical conditions refer to ones’ own belief of the capabilities of vision, hearing and motion in daily lives [69]. With the increase in age, older adults suffer from the gradual loss of sensory and motor systems [70,71]. The decline of physical health conditions hinders their effective usage of ICT systems [72]. Past research has demonstrated the negative relationships between older adults’ perceived health conditions and their perceptions of technology and their intention of technology adoption. For instance, Li, Ma, Chan and Man [73] found that PPC negatively relates to older adults’ perception of the usefulness of health monitoring devices, which in turn lowers their usage intention. PPC is also found to be positively related to perceived ease of use of health informatics systems, which further facilitates older adults’ adoption intention [74]. Ryu, Kim and Lee [64] found that PPC leads to the lower intention of participants in video UGC services.

In order to use VUI effectively, users need to have acceptable health conditions, including visual, auditory, and motion ability. Physical disabilities, such as hearing or speaking problems, can become obstacles for older adults’ effective usage of VUI. The current study targets Chinese older adults who are above 55 years old. These older adults start to experience a decline in physical health conditions, which can possibly influence their perceptions of VUI. Therefore, we expect positive relationships between PPC and PU, PEOU.

**Hypothesis** **H3-1:** *Perceived physical conditions positively influence perceived usefulness*.

**Hypothesis** **H3-2:** *Perceived physical conditions positively influence perceived ease of use*.

#### 3.3.2. Mobile Self-Efficacy

Mobile self-efficacy refers to one’s subjective evaluation of his/her capability to use mobile devices [75]. The UTAUT includes the construct of self-efficacy as a factor to influence PEOU, which further influences adoption intention [31]. Prior research reported that the lack of capacity is one of the difficulties encountered by older adults when learning to use computers [76]. The higher self-efficacy indicates that users have more expertise and abilities in interacting with mobile devices. Self-efficacy is found to be positively related to technology usage [77,78] and older adults’ perception and adoption of geotechnology [60].

In terms of the influence of self-efficacy on users’ adoption of VUI, higher self-efficacy brings about among young people [40,79]. However, high self-efficacy could strengthen users’ attachment to the traditional interaction methods, leading to their resistance to new interaction methods, especially for older adults. In fact, Deng et al. [61] found that older adults often exhibit resistance to change, which further hinders their adoption intention of health information systems. In terms of Chinese older adults’ adoption of VUI in this study, adopting VUI indicates that users need to change their habits, invest considerable learning efforts and spend certain switch costs. For older adults who have high level of mobile efficacy, it becomes even more difficult because of the higher sunk costs, which makes them show more serious resistance to VUI, leading to lower perceptions and trust. The following hypotheses are posited:

**Hypothesis** **H4-1:** *Mobile self-efficacy negatively influences perceived usefulness*.

**Hypothesis** **H4-2:** *Mobile self-efficacy positively influences perceived ease of use*.

**Hypothesis** **H4-3:** *Mobile self-efficacy positively influences trust*.

#### 3.3.3. Technology Anxiety

Technology anxiety mainly refers to the feeling of discomfort that people experience when using technology [80]. It captures the negative emotions while using technologies. According to UTAUT, technology anxiety hinders users’ adoption intention through PEOU [31]. Rendering with the negative emotions, users easily perceive technologies negatively and show resistance to adopting new technologies [60,64,81]. For instance, in the context of using computers, prior research found that technology anxiety makes users fear using computers and making mistakes, leading to fewer possibilities of using computers [82].

In the contexts of older adults’ adoption of VUI investigated in this study, technology anxiety should also have negative influences on their perceptions and trust in VUI. Specifically, although users, in general, may experience technology anxiety to some extent, older adults suffer from it more seriously [83,84,85]. The negative influences of technology anxiety have been found in various contexts, such as older adults’ PU and PEOU of wearable warming systems [86], PEOU of geotechnology [60], adoption intention of mobile health services [61]. Consistent with this line of research, this study hypothesized the similar negative influences of technology anxiety on users’ perception and trust of VUI.

**Hypothesis** **H5-1:** *Technology anxiety negatively influences perceived usefulness*.

**Hypothesis** **H5-2:** *Technology anxiety negatively influences perceived ease of use*.

**Hypothesis** **H5-3:** *Technology anxiety negatively influences trust*.

#### 3.3.4. Self-Actualization Need

Maslow [87] also highlights the need for self-actualization is the highest level of a person’s need. Self-actualization relates to people’s sense of satisfaction, desire for personal growth, and pursuit of actualization personal potential [72]. To pursue self-actualization, people need to be tolerant of new changes, a new phenomenon, and new technologies. People with higher self-actualization needs tend to be more open-minded. They seem to enjoy new adventures through acquiring new skills and making new changes [88]. They would consider using new technologies as an opportunity for fulfilling their self-actualization needs.

In terms of the adoption of VUI among Chinese older adults, self-actualization could serve as a facilitator for their adoption of VUI. The self-actualization need is not only important for early adults but also for older adults. According to Erikson [89], a sense of fulfillment is the ultimate purpose that a person pursues to develop in the later stage in life. Thus, driven by the intrinsic motivation of self-actualization, older adults could view adoption VUI as a chance for new adventures. Previous studies found that self-actualization positively relates to older adults’ adoption of e-government services [72] and wearable health technology [90]. Therefore, in the context of older adults’ adoption of VUI, similar effects were expected.

**Hypothesis** **H6-1:** *Self-actualization need positively influences perceived usefulness*.

**Hypothesis** **H6-2:** *Self-actualization need positively influences perceived ease of use*.

## 4. Research Methods

### 4.1. Sampling and Procedure

To test the proposed conceptual framework, a survey was designed and conducted. A web-based questionnaire was adopted through the professional online platform of ePanel (http://www.research.epanel.cn/, accessed on 8 December 2021). Although online sampling may carry some limitations, it is a valid way for the research aim in this study. The online sampling method was considered as a proper and valid way for data collection in this study because the connection to the Internet and experience with smart devices are required for effective usage of VUI. In other words, users’ experience with the Internet and digital devices is a precondition for VUI adoption. In fact, previous studies have widely used online sampling for investigating users’ adoption of smart devices, such as healthcare wearable devices [91], smart speakers [34], and smartwatches [92].

In terms of participants, participants were included based on two criteria: age and experience with smart devices, such as smartphones, tablets, smartwatches, and smart speakers. As we target older adults, we collected participants who are older than 55 years old, when people’s cognitive and physical capabilities start to decline [93]. The experience with smart devices was also used as a criterion for selecting participants because it is required by effective usage of VUI. If participants had no experience with smart devices, they would have few chances to use VUI.

Participants were first welcomed to this survey and then filled in the consent. Subsequently, participants were asked to fill in their age and experience with smart devices. These two questions served as screening questions. Participants were allowed to continue this survey if they were older than 55 years old and they had experience with at least one smart device. Next, as participants might be unfamiliar with voice interaction technology, participants were presented with a short introduction video, which briefly exhibited the benefits, usage procedures, and usage scenarios of VUI. To specify, this particular VUI was specially designed by a professional interaction designer. The scenario included using VUI to control smart speakers, smartphones and smart televisions. The video was around 90s. After watching this video, participants were asked to indicate their experience with VUI, perceptions and adoption intention of VUI based on a series of statements. Finally, they were asked about their personal information, including demographic information, their perceived physical condition and their psychological characteristics (see Table 1 in the next section).

### 4.2. Measurement

Through the extensive review of current studies, a questionnaire was designed, which included three parts: (1) participants’ perception of voice interaction technology, (2) aging-related characteristics and (3) participants’ demographic information. The measures related to participants’ perception of voice interaction and aging characteristics can be found in Table 1. These measures were based on or adapted from existing validated measures. Participants were asked to indicate their opinions of measures based on a 7-point Likert scale ranging from 1 (strongly disagree) to 7 (strongly agree). The human protocols used in this work were evaluated and approved by Sichuan University (YJ202203).

### 4.3. Data Collection

**Figure 2:**
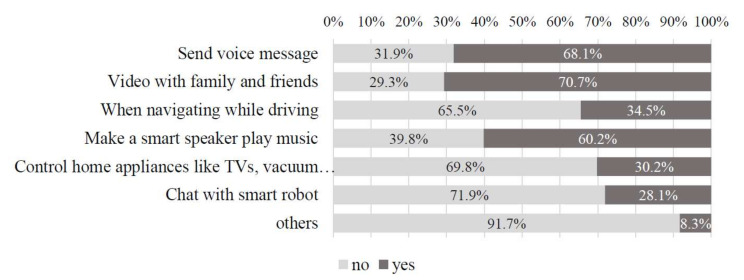
Frequency table of participants’ experience with VUI.

Participants were collected through an online survey. In total, 420 participants were collected (Mean age = 59.67, 50% male). Table 2 showed detailed descriptions of this sample. We aim to cover current and potential users of VUI, which is a commonly used and valid way to investigate users’ adoption intention of specific technologies [91,98].Thus, we did not select participants based on their experience with VUI. We selected participants based on their experience with smart devices, which is a precondition for users’ adoption of VUI. Among the participants who have experience with smart devices, they have experience with VUI more or less. In this way, we capture both current and potential users of VUI. Participants’ experience with VUI can be found in Figure 2.

## 5. Results

The initial descriptive analyses and reliability analyses were conducted by using SPSS 25.0. Next, the data were analyzed by examining the measurement model and the structural model, respectively [99]. AMOS 24.0 was used to conduct the confirmative factor analysis [29] to assess the measurement model and perform path analysis.

### 5.1. Reliability and Validity

The measurement model confirms a goodness of fit: x^2^/df = 1.979, GFI = 0.926, SRMR = 0.040, RMSEA = 0.048, CFI = 0.965, NFI = 0.931. Cronbach’s alpha was calculated for reliability tests. Results revealed satisfactory reliability of all the measures with a threshold of 0.7, except for the measure of perceived physical conditions, which is greater than 0.6 [100]. The AVE values of all the measures are above 0.5, except for PPC. Next, CFA was conducted to assess the validity, including unidimensionality validity, convergent validity and discriminant validity. Results showed that these measures exhibited adequate validity (see Table 3 and Table 4 for details). Specifically, the standardized loadings of all the items are above 0.5. Although most of the average variance extracted (AVE) is above the threshold of 0.5, AVE for PPC is slightly lower than 0.5. Considering that the composite reliability for PPC is higher than 0.6, the convergent validity of the construct is still adequate [101]. As for discriminant validity, the square root of AVE should be higher than the inter-construct correlation in the model; however, some square roots are lower than the correlations (e.g., the relationship among BI, PEOU and PU). Thus, it is necessary to further examine whether the current model achieved satisfactory discriminant validity. The latest research suggests HTMT might be a powerful criterion for discriminant validity assessment [102]. The results of the HTMT (see Table 5) test showed all the values are below the threshold of “0.9”, suggesting it achieved an acceptable discriminant validity. In addition, the composite reliabilities of all the constructs were above 0.7. Taken together, the measures used in this study showed satisfactory validity [103,104].

### 5.2. Structural Model Assessment

Structural equation modeling was used to analyze the proposed research model with AMOS 24.0. The results revealed absolute fit indices and incremental fit indices (see Table 6). All the values are greater than the suggested values [105], which indicates that the data has a good fit with the proposed model and the data is adequate for further path analysis.

### 5.3. Hypotheses Testing and Path Analysis

**Figure 3:**
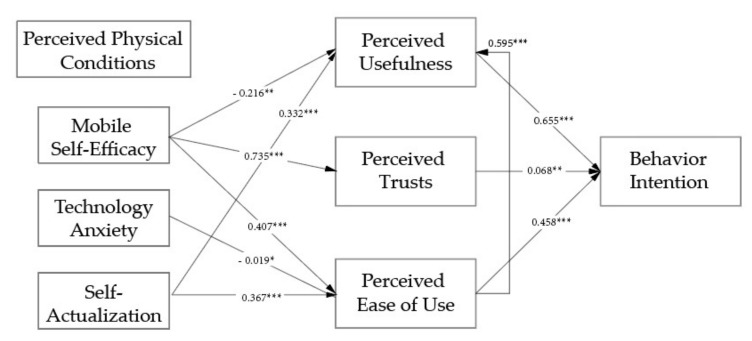
Results of SEM. Note: * *p* < 0.1; ** *p* < 0.05; *** *p* < 0.01.

Path analysis was conducted through SEM to examine the relationships among variables. The results of path analyses can be found in Figure 3 and Table 7. Results revealed that ten out of fourteen hypotheses were supported or partially supported. Behavior intention was predicted by perceived usefulness, perceived ease of use and trust, with a variance of 64.34%. Perceived usefulness was the most determinate variable, followed by perceived ease of use and perceived trust. Moreover, perceived usefulness was predicted by perceived ease of use, mobile self-efficacy and self-actualization, with a variance of 51.09%. Perceived ease of use was explained with the variance of 30.07% by self-efficacy, technology anxiety and self-actualization. Perceived trust was influenced by self-efficacy with a variance of 54.02%.

In terms of the influences of aging characteristics, mobile self-efficacy makes significant influences on perceived usefulness, perceived trust, and perceived ease of use. Technology anxiety influences perceived ease of use negatively in a marginal way. Self-actualization significantly influences perceived usefulness and perceived ease of use.

## 6. General Discussion

Accordingly, the current study tends to contribute to prior literatures in several ways. To begin with, although previous research might have emphasized the introduction of new media and discussed their acceptance towards the latest technology, limited attention has been given to VUI [3], especially in the context of China, one of largest elderly populations in the world [12]. Given the popularity of VUI nowadays, older adults’ adoption intention has been largely overlooked in China. This study addresses the research gap by proposing a model to provide insights on the factors that influence older adults’ adoption of VUI in China. In addition, rare literature has comprehensively discussed the characteristic of VUI and older adults through incorporating the construct of trust and aging-related characteristics (i.e., perceived physical conditions, mobile self-efficacy, technology anxiety, self-actualization). In order to address this gap, this study started from TAM and further extended the model to have a relatively more thoroughly insight of the behavior of the elderly in this digital era. Results revealed that three factors determined Chinese older adults’ adoption of VUI: perceived usefulness, perceived ease of use and trust.

To specify, the results reveal several important findings. Consistent with previous studies on TAM [2]. Findings confirm that perceived usefulness, perceived ease of use, and trust is three important factors to explain Chinese older adults’ adoption of VUI. Results further reveal aging-related characteristics influence older adults’ perception of ease of use, usefulness and trust. This study finds a positive relationship between trust and the adoption intention of VUI. Trust has been demonstrated as an important factor in the contexts of e-commerce, e-government and technology adoption [48,108]. In the context of VUI, as VUI systems need to perform monitoring functions all the time, users have to share their daily conversations with the systems. The exposure of personal information can make users feel uncomfortable and vulnerable, which hinders users’ adoption of VUI. In this case, trust becomes a crucial factor. Users’ belief that their personal information will be protected becomes can largely alleviate users’ negative feelings and facilitate their adoption of VUI. Consistent with prior research that found the role of trust in influencing young adults’ adoption of VUI in the U.S. [40], results of this study show a similar pattern. Users who have a higher degree of trust will have a stronger adoption intention of VUI.

This study also reveals the influences of aging-related characteristics. Among the aging-related characteristics, perceived physical conditions did not show any significant influences on perceived usefulness, perceived ease of use and perceived trust. These findings are consistent with previous studies [61]. One possible explanation would be that healthy conditions serve as a precondition for older adults’ adoption of VUI, but the perceived physical condition itself does not naturally lead to better adoption intention. In other words, relatively healthy physical conditions enable older adults with acceptable physical and cognitive capabilities for using VUI. For instance, a good hearing ability enables older adults to use VUI, but a better hearing ability does not improve their intention of using VUI. Most likely, perceived physical conditions are influenced by other factors, such as technology anxiety.

Different from our hypothesis, no significant influence of technology anxiety is found on perceived usefulness or trust. In line with previous studies [61], technology anxiety lowers older adults’ perception of ease of use of VUI. A marginal significant negative influence of technology anxiety is found on perceived ease of use (*p* < 0.1). This could be influenced by the fact that the benefits of VUI have been well acknowledged by older adults. The anxious emotion does not have a significant influence on their perception of usefulness. Different from other interaction methods (e.g., GUI) that require considerable efforts to acquire, VUI is highly similar to natural speech in daily lives. Such similarities make older adults feel that VUI are close to them and easy to acquire. The anxiety triggered by technology might be largely alleviated because of the intuitiveness of VUI. Thus, no significant influences of technology anxiety on perceived usefulness or trust were detected.

In terms of mobile self-efficacy, as expected, it positively affects perceived ease of use and trust. The extensive experience with mobile devices provides users with a better capability of learning VUI, and thus, they have a more positive perception of ease of use. Similarly, their experience with other technological applications, such as e-commerce, also translates into higher trust with VUI. Through their previous experience, they understand that technology provides have the obligation to protect users’ personal information. There are laws and rules to prohibit the misuse of users’ personal information. Therefore, older adults who have a higher level of mobile self-efficacy form a higher degree of trust with VUI. However, the higher level of self-efficacy does not bring a higher perception of usefulness. Instead, high self-efficacy is found to lower older adults’ perception of the usefulness of VUI. This finding indicates that older adults with a higher level of self-efficacy have more serious resistance to VUI. Specifically, older adults who are skillful at traditional interaction methods may feel that the traditional ways can satisfy their needs and it is unnecessary to change into VUI. Consequently, they have a negative perception of the usefulness of VUI.

As for self-actualization, consistent with our hypotheses, it positively relates to perceived usefulness, perceived ease of use, and perceived trust. Self-actualization is an intrinsic motivation to make achievements [87]. In line with previous studies that show that a higher level of self-actualization is associated with older adults’ adoption of new technologies [61,72,109], this study further confirms this notion by revealing the positive relationship between a higher level of self-actualization and the perception of VUI. Chinese older adults view using VUI as a chance for personal development.

### 6.1. Practical Implications for Facilitating VUI Adoption

Chinese older adults’ adoption of smart devices remains relatively low [110]. The complicated interaction is one of the barriers to older adults’ effective usage of smart devices. Using VUI as an interaction method could be a chance to assist older adults’ effective usage of smart products. This study finds that older adults’ adoption of VUI is predicated by perceived usefulness, perceived ease of use and trust. These factors also serve as mediators for the influences of technology anxiety, mobile self-efficacy and self-actualization on older adults’ adoption of VUI. These findings have valuable implications for developers and promoters to develop better VUI and plan for better communication strategies to facilitate adoption by older adults.

Developers should improve the speech recognition quality and language processing quality of VUI, as older adults show a higher adoption intention when they perceive VUI as more useful and ease of use. Both usefulness and ease of use of VUI rely on speech recognition accuracy and natural language processing capability. The higher accuracy of users’ voice commands and better comprehension of users’ intended meanings further improve VUI’s usefulness and ease of use. Specifically, for improving perceived usefulness, developers should carefully assess the contexts for using VUIs. The usage of VUIs can be particularly helpful for complex interaction tasks that require multiple steps, such as searching and navigation tasks. It would be also useful for using VUIs in tasks that are difficult for older adults due to decreasing capabilities, such as typing and dialing tasks.

To improve users’ perception of ease of use, developers can also make the voice interaction simple and intuitive. Involving interpersonal communication techniques into VUI can be particularly helpful for older adults. Designers can think of creating a personality for VUIs, which can largely reduce the psychological distance perceived by older adults. Designers should carefully consider how to create a desirable personality, including gender, tone, speaking styles. As older adults suffer from reduced cognitive load, it would be helpful to use short vocabularies that are easy to remember, such as ‘OK’ and ‘got it’. When it is necessary to highlight certain information, it would also be useful to slow down the speed and improve the volume of voice commands.

Moreover, it is important to improve trust between older adults and VUI. Developers could explore new technologies solutions to improve privacy when using VUI. When promoting VUI, marketers could highlight the sophisticated technologies used to improve privacy as well as the agreements with users for protecting users’ personal information. Policymakers could also try to explain the regulations in law for protecting users’ information and the serious consequences for the misuse of users’ personal information.

This study further shows the influences of mobile self-efficacy, technology anxiety, and self-actualization, which are useful for developers and marketers. Older adults who have a higher level of mobile self-efficacy show a higher perception of ease of use and trust, but a lower perception of the usefulness of VUI. This indicates that a higher level of mobile self-efficacy makes older adults more resistant to the benefits of VUI. When promoting VUI, marketers need different communication strategies for older adults who have a low or high level of mobile self-efficacy. It is necessary to highlight the benefits of VUI, especially the relative advantages of VUI in comparison with previous interaction methods. It would be also possible to first target older adults who have a low level of mobile self-efficacy. Moreover, it seems that VUI is an intuitive interaction method and thus, the influence of technology anxiety is relatively limited. Technology anxiety is found to be marginally related to perceived ease of use negatively. Therefore, developers and markers do not need to pay extensive efforts on how to reduce technology anxiety. In addition, self-actualization is found to make positive influences on perceived ease of use, perceived usefulness, and trust. This finding indicates that marketers should express the message that using VUI is a channel for personal development. Marketers could use multiple channels to express these messages, such as short videos on social media and graphic posters in public places. These efforts could facilitate older adults’ adoption of VUI.

### 6.2. Practical Implications for Using VUI in Smart Home Systems

Older adults show resistance to adopting smart home devices although they can gain huge benefits from adopting smart home systems. The integration of VUI in smart home systems is promising to facilitate older adults’ adoption of smart home systems. The results of this research not only provide implications for older adults’ adoption of VUI but also for their adoption of smart home systems.

For developers, when integrating VUI into smart home devices, they should pay particular attention to users’ perception of ease of use and usefulness. Specifically, for some smart products, such as smart speakers, the integration of VUI can largely improve users’ perception of ease of use and usefulness because smart speakers provide various functions which require complex interactions. In this case, integration of VUI largely reduces older adults’ learning burdens, which improves their perceptions of ease of use and usefulness of smart speakers in general. Differently, for some products that require simple interactions, integrating VUI may not be an optimal choice because the improvements of perceptions of usefulness and ease of use remain limited. For instance, for a cleaning robot whose function is to clean floors autonomously, users interact with it by pressing a start button, which is direct and simple. Upon completion, users have to physically interact with it in order to clean the dust containers. Thus, because of the simple interaction and requirements of physical interaction, involving VUI in cleaning robots might not largely improve users’ perception of ease of use and usefulness. As developing and integrating VUI into smart devices is costly, developers should carefully consider the appropriateness of involving VUI in smart devices.

This study also shows the influences of aging-related characteristics on older adults’ adoption of VUI, which could also be applicable to explaining their adoption of smart home devices. Specifically, mobile efficacy may lower users’ perceptions of the usefulness of smart home devices, similar to users’ perceptions of VUI. Because users who are very familiar with current mobile devices may feel that these devices sufficiently satisfy their needs, it is not necessary to switch to smart devices. Therefore, to promote older adults’ adoption of smart home devices, it would be interesting to highlight the benefits provided by smart home devices and target users who are less familiar with mobile devices.

In addition, we found a positive relationship between self-actualization and adoption of VUI. It is possible that self-actualization also positively influences older adults’ adoption of smart home devices. When older adults have a higher level of self-actualization, they are more motivated to adopt VUI because they view learning VUI as a chance for personal development. Similarly, for older adults with high self-actualization, learning to use smart home systems could also become an opportunity for them to gain new experiences. Thus, to promote smart home devices, companies should highlight self-actualization messages and target older adults who have a relatively high level of self-actualization.

### 6.3. Limitations and Future Research

Although this study is carefully prepared, it carries several limitations. We conducted the data collected online. According to CNNIC, 70% of older adults in China are frequent users of the Internet and mobile Internet [110]. The adoption of smartphones exceeds 80%. The high penetration rate of smartphones and the Internet makes it feasible to collect data online. As VUI is often integrated with smart products, it is also suitable to use the online sampling method. However, the older adults who are less active online might not be covered in this sample. In other words, whether these results can be applicable for older adults who are not frequent users of the Internet still requires further validation, which can be interesting for future research. Moreover, this study provides evidence on the potential usage of VUI toward the target population. A future study can use field experiments to validate the current finding. Specifically, it would be interesting to collect elderly participants who have some hands-on experience regarding VUI usages, which can result in more specific guidelines for developing usable VUI for older adults.

In addition, the average age of participants is 59, who are labeled as young old adults. This group of older adults occupies a large proportion in China, and thus it is worthwhile to focus on this group. However, this group of older adults could be different from older adults whose ages exceed 65. Therefore, future research could replicate this study by focusing on older adults with higher ages. Moreover, this study focuses on VUI adoption intention and older adults’ general perception of VUI. In other words, although older adults are willing to adopt VUI in their daily lives, their actual usage and continuous usage remain unknown. Older adults’ actual usage might be influenced by other factors, such as usability and usage scenarios. Future research could conduct user studies to learn the usability issues with using VUI and generate guidelines for VUI development, which can further facilitate the adoption of VUI.

## 7. Conclusions

VUI has gained popularity in this decade. It has been integrated with various smart home devices and developed for many usage scenarios. The benefits of VUI should be available to everyone, including older adults, who occupy 25% of the overall population in China. This study investigates the factors that influence older adults’ adoption of VUI in China. On the basis of TAM, this study proposes a theoretical model to predict older adults’ adoption of VUI through incorporating the construct of trust and aging-related characteristics (i.e., perceived physical conditions, mobile self-efficacy, technology anxiety, self-actualization). A survey was conducted with 420 participants who are current or potential users of VUI. Data were analyzed through SEM and the data showed a good fit of the proposed theoretical model. Results further revealed that older adults’ adoption is determined by perceived usefulness, perceived ease of use and trust. These factors also mediate the influences of aging-related characteristics on older adults’ adoption of VUI. Specifically, mobile self-efficacy is found to make positive influences on trust and perceived ease of use, but negative influences on perceived usefulness. Self-actualization makes positive influences on perceived usefulness and perceived ease of use. Technology anxiety only exerts a marginally significant influence on perceived ease of use. No significant influences of perceived physical conditions were found. These results extend the TAM and STAM by incorporating additional variables. These results also provide valuable implications for practice.

## Figures and Tables

**Table 1:** Constructs and measurements.

Construct	Measurement Item	References
Behavior Intention (BI)	BI1: I predict I would use voice interaction in my smartphone to conduct tasks.	[94]
	BI2: In the future, I will often use voice interaction to manage my smartphone.	
Perceived Usefulness (PU)	PU1: I think that using a voice interface increases productivity.	[95,96]
	PU2: I think that a voice interface is useful.	
	PU3: Using a voice interface would make my life convenient.	
Perceived Ease of Use (PEOU)	PEOU1: It would be easy for me to become skillful at using a voice interface.	[95]
	PEOU2: It would be easy for me to use voice interaction in the way I like to.	
	PEOU3: Leaning to use voice interaction is entirely within my capability.	
Trust (TRU)	TRU1: I trust that my personal information will not be used for any other purpose.	[40]
	TRU2: I believe that my personal information is protected.	
	TRU3: I am assured that my information is secure.	
Perceived Physical Conditions (PPC)	PPC1: How is your hearing?	[97]
	PPC2: How is your vision?	
	PPC3: How is your mobility ability?	
Mobile Self-Efficacy (SE)	SE1: I am fluent in the use of a mobile device.	[75]
	SE2: I can figure out almost any mobile application with a minimum of effort.	
	SE3: I feel I am able to use the mobile internet to browse the world wide web.	
Technology Anxiety (TA)	TA1: Using voice interaction would make me very nervous.	[31]
	TA2: Using voice interaction would make me worried.	
	TA3: Using voice interaction would make me feel uncomfortable.	
	TA4: Using voice interaction would make me feel uneasily unconfused.	
Self-actualization needs (SA)	SA1: Learning to use voice interaction increases my feeling of self-fulfillment.	[72]
	SA2: Learning to use voice interaction gives me a feeling of accomplishment.	

**Table 2:** Descriptive analysis of participants.

Characteristics		Frequency	Percentage (%)
Age	55–59	216	51.4%
	60–64	160	38.1%
	65–69	30	7%
	Above 70	14	3.5%
Gender	Male	210	50%
	Female	210	50%
Education	Elementary	7	1.7%
	Junior High School	51	12.1%
	High School	134	31.9%
	College/university	216	51.4%
	Postgraduate	12	2.9%
Income	Below 50 k RMB	63	15%
	50 k–10 k RMB	117	27.9%
	10 k–15 k RMB	88	21%
	15 k–20 k RMB	88	21%
	20 k–30 k RMB	52	12.4%
	Above 30 k RMB	12	2.9%

**Table 3:** Reliability and unidimensionality.

Construct	Variables	Cronbach’s Alpha	Standardized Loading	C.R/t-Value.	AVE	Composite Reliability
BI	BI1	0.786	0.813	-	0.649	0.787
	BI2		0.798	17.376		
PU	PU1	0.755	0.742	-	0.509	0.756
	PU2		0.705	13.035		
	PU3		0.692	12.814		
PEOU	PEOU1	0.747	0.733	-	0.547	0.783
	PEOU2		0.747	12.648		
	PEOU3		0.739	12.549		
TRU	TRU1	0.875	0.875	-	0.708	0.879
	TRU2		0.768	18.5		
	TRU3		0.877	22.132		
PPC	PPC1	0.641	0.753	-	0.444	0.700
	PPC2		0.703	6.286		
	PPC3		0.521	5.637		
SE	SE1	0.833	0.752	-	0.633	0.838
	SE2		0.828	16.006		
	SE3		0.806	15.687		
TA	TA1	0.950	0.926	-	0.826	0.950
	TA2		0.921	32.861		
	TA3		0.883	29.25		
	TA4		0.905	31.302		
SA	SA1	0.763	0.733	-	0.623	0.767
	SA2		0.842	14.013		

**Table 4:** Constructs correlation matrix.

	PPC	SE	TA	SA	TRU	PU	PEOU	BI
**PPC**	**0.667**							
**SE**	0.499	**0.796**						
**TA**	−0.009	0.153	**0.909**					
**SA**	0.360	0.411	−0.086	**0.789**				
**TRU**	0.378	0.563	0.105	0.610	**0.841**			
**PU**	0.340	0.347	−0.177	0.734	0.439	**0.713**		
**PEOU**	0.441	0.697	−0.057	0.658	0.672	0.683	**0.740**	
**BI**	0.405	0.523	−0.115	0.764	0.610	0.853	0.840	**0.806**

**Table 5:** The HTMT Analysis of discriminate validity.

	PPC	SE	TA	SA	TRU	PU	PEOU	BI
**PPC**	**-**							
**SE**	0.556	**-**						
**TA**	0.015	0.153	**-**					
**SA**	0.405	0.403	0.081	**-**				
**TRU**	0.415	0.575	0.096	0.644	**-**			
**PU**	0.397	0.340	0.177	0.720	0.437	**-**		
**PEOU**	0.520	0.726	0.058	0.693	0.729	0.712	**-**	
**BI**	0.444	0.525	0.115	0.757	0.622	0.852	0.881	**-**

**Table 6:** Goodness-of-fit test.

Category	Measure	Acceptable Values	Value
Absolute fit indices	Chi-square/d.f.	1–5	2.248
	GFI	0.90 or above	0.913
	SRMR	0.08 or below [106]	0.065
	RMSEA	0.08 or below [107]	0.055
	NFI	0.90 or above	0.920
Incremental fit indices	IFI	0.90 or above	0.954
	TLI	0.90 or above	0.942
	CFI	0.90 or above	0.953

Note: GFI = goodness-of-fit index; SRMR = standardized root mean square residual; RMSEA = root mean square error of approximation; NFI = normed fit index; IFI = incremental fit index; TLI = Tucker–Lewis index; CFI = comparative fit index.

**Table 7:** Results of hypotheses testing.

	Path Direction	Path Coefficients	*p*-Value	Results
H1-1	PU → BI	0.655	***	Supported
H1-2	PEOU → BI	0.458	***	Supported
H1-3	PEOU → PU	0.595	***	Supported
H2	TRU → BI	0.068	0.028 **	Supported
H3-1	PPC → PEOU	0.015	0.209	Not supported
H3-2	PPC → PU	0.078	0.277	Not supported
H4-1	SE → PEOU	0.407	***	Supported
H4-2	SE → PU	−0.216	0.005 **	Supported
H4-3	SE → TRU	0.735	***	Supported
H5-1	TA → PEOU	−0.019	0.090 *	Partially supported
H5-2	TA → PU	−0.015	0.188	Not supported
H6-1	SA → PEOU	0.367	***	Supported
H6-2	SA → PU	0.332	***	Supported

Note: * *p* < 0.1; ** *p* < 0.05; *** *p* < 0.01.

## Data Availability

The data used in this study are available upon request from the corresponding author.
